# Sensory and developmental phenotyping of *C. elegans* parses autism associated genes into behavioural classifications

**DOI:** 10.64898/2026.03.27.714775

**Published:** 2026-03-30

**Authors:** James W Lamb, Eleonora M Pieroni, Farah Al Khawaja, Katrin Deinhardt, Vincent M O’Connor, James C Dillon

**Affiliations:** 1 School of Biological Sciences, University of Southampton, Southampton, SO17 1BJ, United Kingdom.; 2 Department of Cell Biology, University of Bremen, 28334, Bremen, Germany.

## Abstract

A large subset of ASD associated genes, almost 50% of the highest confidence risk genes listed on the Simons Foundation Autism Research Institute database, are epigenetic modifiers. This suggests that the organization of sensory biology and its coupling to underlying genetic control are an important element underpinning this discord. Furthermore, sensory processing changes in individuals with autism spectrum disorder (ASD) has been a growing area of study in recent years. *C. elegans* have robust readouts for both developmental and sensory biology allowing these signatures of ASD to be systematically modelled.

52 epigenetic modifiers (65 strains) were selected for study in *C. elegans* based on gene function, presence of orthologues in *C. elegans* and the availability of viable putative null strains. This highlighted significant changes to reproduction, gross development and sensory processing across the range of epigenetic modifiers. Each strain was filtered against selective criteria for significant sensory and developmental phenotypes allowing for selective phenotypic profiles to emerge. These were three primary groups, those with sensory perturbations but unaffected gross development (6), developmentally affected genes with intact sensory function (10) and finally genes with impaired gross development and sensory function (11). Thus, this study provides a link between sensory and developmental outcomes in ASD associated mutant strains and suggests that more regular sensory testing should be performed in human cohorts to further refine sub-categorisation of ASD cohorts.

## Introduction

Autism spectrum disorder (ASD) is a pervasive developmental disorder clinically identified by two behavioural features encompassing social communication/interaction and restricted/repetitive patterns of behaviour (American Psychiatric Association, 2013). These behavioural traits manifest during early development and then persist with varying levels of severity (American Psychiatric Association, 2013). In addition, perturbation of sensory processing in ASD cohorts has been observed across multiple modalities (Balasco et al., 2020) and been weighted as an important trait although this has not been systematically coupled to other clinical measurements that have raised discrete routes to ASD. Sensory testing ranges from parent’s self-reporting behaviours (Balasco et al., 2020) to matched cohort sensory testing in laboratory conditions (Balasco et al., 2020).

The genetic architecture of ASD is becoming better understood, and the increasing quantitative nature of these investigations allows genotype and associated traits to be grouped opening up description to inform on mechanism. With heritability rates ranging from 75%–95% (Colvert et al., 2015), the contribution of genetic mutation to the aetiology of ASD is dominant but is unable to provide a wholistic explanation of the disorder. A cornerstone of this is rooted in the abundance of genetic determinants associated with ASD discovered through genome wide association studies (GWAS) (Grove et al., 2019), whole exome sequencing (WES) (Yu et al., 2013) and twin studies (Tick et al., 2016). The weight of these identified genetic risk factors are catalogued and ranked on the Simons Foundation Autism Research Initiative (SFARI) gene database with over 1200 (Jan 2026) (Abrahams et al., 2013) genes implicated in the aetiology of the disorder.

This is likely complicated by the now established environmental cause that is mediated by secondary regulation of the genome. The most likely candidate for environmental-genome interactions is through epigenetic modifiers which are found abundantly in the SFARI gene database (Abrahams et al., 2013). This makes these genes particularly interesting to study in the aetiology of ASD as they modulate the environmental impact and have broad transcriptional regulation consistent with context dependent outputs and are likely to underpin the spectrum observed in ASD.

The intersection between genetics, development and environment will enable classifications that parse the autism diagnosis into distinct sub-cohorts. A recent study has validated that ASD cohorts can be statistically parsed by combining phenotypic presentation of clinical symptoms including developmental milestones, repetitive behaviours, social communication and child behaviour, and associated genetic signature (Litman et al., 2025). The four key groups were sub-categorised as moderate challenges, broadly affected, social and/or behavioural and mixed ASD with developmental disorder (DD). Findings from another study seeking to investigate possible subgroupings of Autism cohorts found three subgroups of autistic individuals (Leyhausen et al., 2025). These results were correlated based on imaging phenotypes and transcriptomic profiles (Leyhausen et al., 2025).

Interestingly they are built without clear use of the association of neuroatypical sensory sensitivity. Hyper or hyporeactivity to sensory input is commonly observed in individuals with ASD, it has been postulated to originate in altered neural pathways (Palau-Baduell et al., 2012), atypical sensory modulation (Giacardy et al., 2018) and sensory gating dysfunction (Chien et al., 2019). A definitive explanation for sensory perturbations has yet to be uncovered but has excellent potential to refine the emerging cohorts.

Based on this background we wanted to use *C. elegans* to investigate the intersection between environmental and genetic determinants that impact on sensory signalling that might underpin facets of autism. *C. elegans* has the advantage of genetic tractability along with a diverse range of quantitative assays available in which the environmental cues that drive sensory sub-behaviours can be quantified.

Here we use quantified *C. elegans* development and sensory responses across autism associated gene expression regulators. This pipeline readily developed scores of development and integrity of sensory processing that described strains by their distinct grouping. The groupings showed “sensory”, “developmental” or combined “development, reproduction and sensory” scores relative to N2. This outcome is consistent with the value of driving analysis through sensory assays, and it promotes better collation of such measures in human cohorts.

This outcome will hopefully platform quantitative investigation of gene expression to better define if distinct molecular changes converge on discrete behavioural subcategories that may inform clinical investigation of ASD.

## Materials and Methods

### *C. elegans* strains and culturing

The N2 (Bristol Strain) was obtained from the *Caenorhabiditis* Genetics Center (CGC, University of Minnesota, USA. Funded by NIH Office of Research Infrastructure Programs (P40 OD010440)). The remaining strains were obtained from the CGC and National Bioresource Project and made by S. Mitani (NBRP) (Yamazaki et al., 2010) (Table S1). All strains were cultured at 20°C on standard nematode growth medium (NGM). The worms were maintained on *Escherichia coli* strain OP50 (Brenner, 1974).

### Bioinformatics

Category 1 and category S genes listed on SFARI (Accessed, Jan 2026, Ver 2025 Q4, https://gene.sfari.org/), were the basis for candidate gene selection. Gene expression modifiers were identified by review of the literature using the descriptor ‘gene expression modifying activity’ as the criteria to functionally annotate the genes selected from the SFARI database. The key GO descriptors were ‘transcriptional regulation’, ‘translational regulation’ and ‘regulation of gene expression’. Ortholist2 (http://ortholist.shaye-lab.org/) (Kim et al., 2018) and STRING (https://string-db.org/) (Szklarczyk et al., 2023) were used to determine whether *C. elegans* contains an orthologue of the gene expression modifiers identified in the previous step. Viable mutants were identified using Wormbase (Version: WS297; https://wormbase.org/species/c_elegans#401−-10) which contains JBrowse2 for strain identification and reported phenotypic information on available strains such as the effect of the mutation allowing for those with a high variant effect predictor score (VEP). This enabled exclusion criteria such as sterility or lethality to be applied to each candidate gene strain. Strains identified were purchased from the CGC or NBRP. Additional information on human expression of candidate genes was collated using Bgee (https://www.bgee.org/) (Bastian et al., 2021) and *C. elegans* transcript number data using CeNGEN (https://cengen.shinyapps.io/CengenApp/) (Taylor et al., 2021). Sequence alignments of amino acids for identity and similarity comparison of orthologues was performed with EMBOSS Needle (https://www.ebi.ac.uk/Tools/psa/emboss_needle/) (Madeira et al., 2024).

### Egg Laying and Development Analysis

A synchronised population of hermaphrodite worms was generated by allowing 3 L4+1-day (reproductive young adults) worms to lay eggs for 3 hours before removal. This was then converted to an egg laying rate by dividing by the number of worms and time allowed for egg laying. Developmental analysis was performed using the same plates with which egg laying was recorded by allowing the eggs to develop in culture incubated at 20°C. At set time points, 0 hours, 20 hours, 25 hours, 43 hours, 48 hours and 67 hours the plates were observed under a stereo microscope (Nikon SMZ800N) to count the number and developmental stage of the worms. These stages are defined as either eggs, L1 or L2 for early development and L3, L4 and young adults for late development as described (Thummel, 2001). All strains were compared to N2 and the distribution and variation of the distinct stages used to determine the developmental timings. Additionally, *che-3 (e1124)* was included to benchmark sensory deficits on development in *C. elegans*. Strains were rank ordered for developmental delays vs N2 and a correlation of early and late developmental rank orders for each strain was performed. Experimenters were blind to the strains being investigated during the assay and statistical analysis.

### Confocal Microscopy and DiI Labelling

A synchronised population of L4+1-day (72 hours old) worms were washed once with 1 ml M9 before being resuspended in 1ml DiI in M9 (2mg/ml Invitrogen, Catalogue: D282) and left on a shaker for 3 hours at room temperature. Worms were washed 3 times post staining and re-plated onto an OP50 seeded NGM plate for 15 minutes prior to imaging. Individual worms were picked into single drops of 10mM sodium azide (CAS 26628–22-8, Sigma-Aldrich) solution on microscope slides containing 1% agar pads to paralyse them for imaging (Schultz and Gumienny, 2012). Worms were then imaged using a Leica DMI8-CS microscope with excitatory laser DPSS 561 nm and collection of emitted light at 565 nm – 660 nm. Following staining, sensory amphids ASK, ADL, ASI, AWB, ASH and ASJ and phasmids PHA and PHB were identified using their anatomical location and morphology (Andrews et al., 2025). The above protocol successfully stained the 6 amphids and 2 phasmids in all N2 worms. N2 staining was run in parallel to the experimental strains and was used as a reference to assess the number and morphology of their sensory neurons. Strains were deemed to be significantly different if at least one amphid or phasmid did not label. Experimenters were blind to the strains being investigated during the assay and image analysis.

### Quadrant Assay

The arena consisted of a 6cm petri dish with 13ml NGM. On the plastic base of the dish the plate was split into 4 equal sized quadrants. A dot was placed in each quadrant 2 cm from the intersect point of the quadrant lines. An immobile worm zone was also outlined at 0.5 cm from the intersect point in all directions. The quadrants were split into 2 test and 2 control diagonally mirrored. On the day of the assay, 1 μl 0.5 M sodium azide was pipetted on the dot in each quadrant first and allowed to dry. This was followed by either 1 μl M9 in the control quadrants or 1 μl 1% v/v diacetyl (Sigma-Aldrich, Catalogue: 8.03528) in M9. A synchronised population of worms was generated by allowing 7 L4+1-day worms to lay eggs for 4 hours. The population of L4+1-day worms generated were washed twice using 1ml M9 solution before being transferred to the assay plate with a glass capillary onto the intersect point of the quadrant in <15 μl M9. The assay plates were then left for 50 minutes and scored based on the number of worms in each quadrant. A chemotaxis coefficient was obtained by taking the differential of worms on the test vs control quadrants divided by the total number of worms in the assay. Worms still within 0.5 cm of the start point were counted as non-moving and the percentage of non-moving worms per mutant was recorded and compared to N2. Experimenters were blind to the strains being investigated during the assay and statistical analysis.

### Droplet Avoidance Assay

We adapted the classical acute aversion assay first described by Hilliard et al., 2002; Hilliard et al., 2004. 9 cm plates were poured with NGM (20ml) three days prior to the assay. 7 L4+1day old worms were picked onto an unseeded NGM plate and allowed to roam for 30 seconds to remove residual bacteria. These worms were then transferred onto a fresh unseeded 9 cm NGM plate and left for at least 20 minutes to allow the transition from local area search to escape behaviour (Gray et al., 2005). One drop (<20 μl) of the indicated aversive cue was placed on the trajectory of a forward moving worm. Soluble aversive cues used in this study included copper sulphate (CuSO4, 30mM), M9 (pH 3.0), and fructose (4M) in water and were controlled against M9 (pH 7.0). The volatile aversive cue used was 30% v/v 1-octanol in ethanol and presented on a platinum wire in the trajectory of a forward moving worm.

The performance of the selected autism associated gene mutant strains was recorded and compared to the N2 and a classically defined sensory mutant *che-3 (e1124*) (sensory deficient control). *che-3 (e1124)* lacks all sensory amphids and phasmids (Wicks et al., 2000). Video recordings were made of the worm’s response to the sensory cue and analysed *a posteriori*. For soluble aversive cues (water soluble cues), the total number of reversals initiated within 5 seconds of compound exposure was counted. For the volatile aversive cue 1-octanol (30% v/v), the latency from cue introduction to initiating a reversal was recorded. Experimenters were blind to the strains being investigated during the assay and video analysis.

### Significance Heatmap and phenotypic criteria

Data from statistical tests of each ASD associated mutant in all assays conducted were compiled. Statistically determined p values for each strain, from post-hoc multiple comparison tests, in every condition (excluding DiI labelling) were compiled into a single database. From this dataset each p value was converted into a binary value of 1 or 0 for significant (p<0.05) or non-significant (p>0.05) respectively. These binary values were then plotted to show each individual strains significant perturbations in each assay compared to N2. Developmental delay was defined as deviating significantly from N2 in either early or late development. Sensory impairment was defined as deviating significantly from N2 in either attractive or aversive (soluble or volatile) sensory processing. Severe sensory impairment was defined as significantly deviating from N2 in both attractive and aversive (soluble or volatile) sensory processing.

### Statistical Analysis

Statistical analysis was performed using GraphPad Prism 10.4.2. All data expressed as mean ±SEM. Statistical tests and post-hoc analysis are outlined in figure legends. Significance defined at p<0.05.

## Results

### A bioinformatic pipeline for selection of ASD associated gene expression modifiers: comparative phenotypic analysis in *C. elegans*

The SFARI database was used and those listed as category 1 genes were selected for the current pipeline. Our intention was to bias the investigation to select for genes that encode modulators of gene expression.

**Fig 1.**
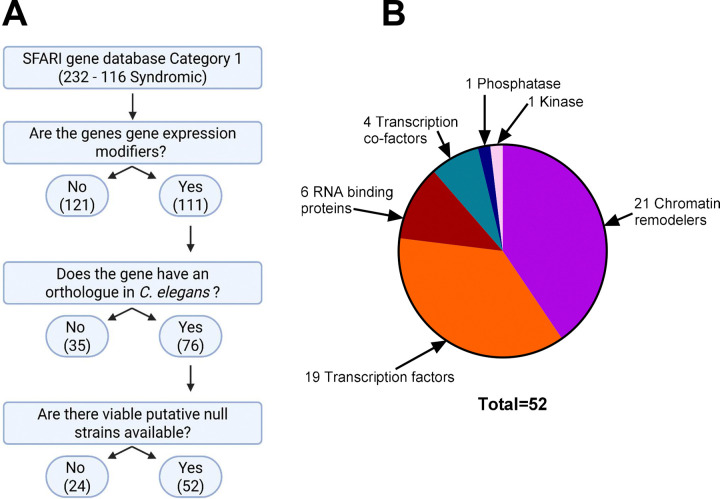
Schematic process and diagrammatic outcome of selected ASD-linked gene expression modifiers conserved in *C.elegans* **A** Schematic diagram showing the bioinformatic filtering process of ASD related gene expression modifiers found in the SFARI gene database. Numbers in brackets are the number of genes for each group. Created in BioRender. Lamb, J. (2026) **B:** The breakdown of the 52 candidate genes based on their molecular function. Includes: chromatin remodelers, RNA binding proteins, transcription co-factors, phosphatase, kinase, and transcription factors. Molecular function was defined by primary literature search or gene ontology (GO) with additional verification using available structural motif

Of the 232 category 1 genes, 111 (47.8%), met the indicated definition of being gene expression modifiers (See Methods). Orthologues were identified using Ortholist 2 (Kim et al., 2018) and STRING (Szklarczyk et al., 2023). Further criteria refined the candidate gene pool to those with an orthologue in *C. elegans* (Fig 1A, 76 genes).

*C. elegans* strains with putative null mutations in orthologous genes were identified (Supplemental Table 1). Mutant alleles were either large deletions or point mutations with a high variant effect predictor (VEP) score (Szklarczyk et al., 2023) indicating the probable loss of protein function by instability or nonsense mediated decay. Mutant strains for 52 (68.4%) of the 76 orthologous genes identified were selected for further downstream analysis. Whilst mutant strains were available for the other 24 orthologous genes, they were not selected based on the mutation generating lethal or sterile strains being unsuitable for our downstream phenotypic analysis. In the case of some genes, up to two strains with distinct alleles were analysed.

Each gene was assigned based on molecular function identified from primary literature searches for the human orthologue. The molecular function of the 52 candidate genes can be classified into two primary groups; chromatin remodelers and transcription factors which make up 40% and 35% of the total respectively (Fig 1B). RNA binding proteins and transcription co-factors contribute 13% and 8% of the total. Finally, a single phosphatase and kinase emerged through the selection pipeline described above. These were included in this analysis despite not directly driving transcription or translation functions due to phosphorylating or dephosphorylating gene expression modifiers (Atas-Ozcan et al., 2021; Smolen et al., 2023).

### Experimental pipeline for the phenotypic analysis of selected *C. elegans* mutants

**Fig 2.**
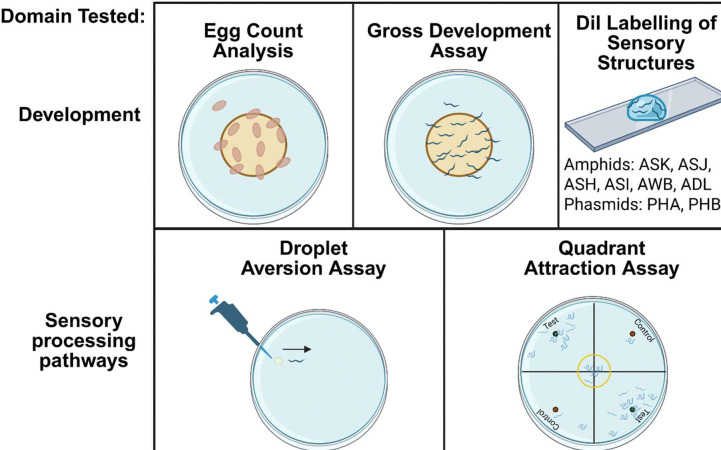
Assay schematics for interrogating the developmental and chemosensory capabilities of *C. elegans* Assay schematics of assays used within this pipeline to interrogate the developmental and chemosensory phenotypes of *C. elegans* strains with ASD associated mutations. For a readout of reproduction an egg count analysis was used. For determining developmental capabilities a gross developmental assay and DiI labelling of sensory amphids and phasmids were used. For determining sensory capabilities, a droplet aversion assay (aversive chemosensory) and quadrant attraction

A combination of developmental, anatomical and sensory driven behavioural assays were conducted on the selected *C. elegans* strains (Fig 2). These were based on robust assays which have already been validated for measuring either sensory or developmental phenotypes (Hilliard et al., 2002; Bargmann, 2006; Altun, 2009). Sensory assays were performed using the aversive cues low pH, fructose, copper sulphate and octanol, whilst diacetyl was used as an attractive cue. These are established sensory cues known to drive behavioural responses in *C. elegans* that can be readily quantified.

### ASD associated mutants show significant disparate effects on egg laying

**Fig 3.**
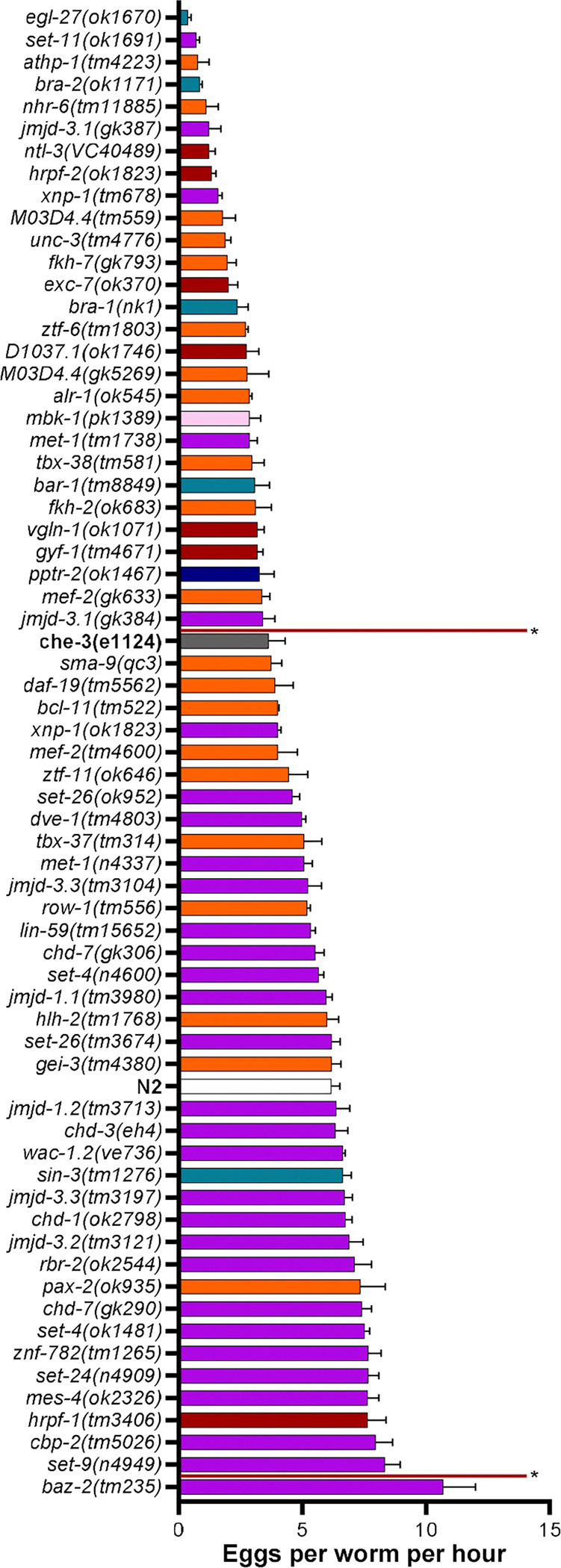
Egg count analysis of N2 vs ASD associated mutants. Egg count analysis comparing the egg laying rate of 3 worms over 3 hours between N2, ASD associated mutants and chemosensory deficient mutant *che-3 (e1124)*. All data shown as mean±SEM Statistical analysis performed using one-way ANOVA with Dunnetts’s multiple comparison test. n = 41 for N2 and n = 3 for all other mutants. Vertical red lines indicate significance compared to N2, strains not contained within the red lines are significantly different. Chromatin remodelers, transcription factors, RNA binding proteins, transcription co-factors, phosphatase, kinase.

28 mutants (43%) spanning all the functional subgroupings had a significantly lower egg laying rate than N2 (Fig 3). The lowest egg laying rate was observed for the mutant *egl-27(ok1670)*, a phenotype that has been previously described (Trent et al., 1983). Only one mutant, *baz-2(tm235)* encoding a chromatin remodeller had a significantly higher egg laying rate compared to the N2. Interestingly, chromatin remodellers were the least affected group with only 6 strains (19.4%) showing a significant difference compared to N2. By contrast, 57.9% of transcription factors and 85.7% of RNA binding proteins. This assay can therefore highlight molecular functional specific phenotypes based on the integrative behaviour of egg laying, suggesting dysregulation of reproductive systems is more likely to have a negative impact on fecundity. Additionally, although sensory processing including detection of food cues impact this behaviour, this reduction in egg laying is not easily explained by perturbations in sensory processing. This is evidenced by the fact that the chemosensory deficient mutant *che-3 (e1124)* egg laying rate was not significantly reduced (Fig 3).

### Pervasive and transient developmental delays observed in ASD associated mutants

**Fig 4.**
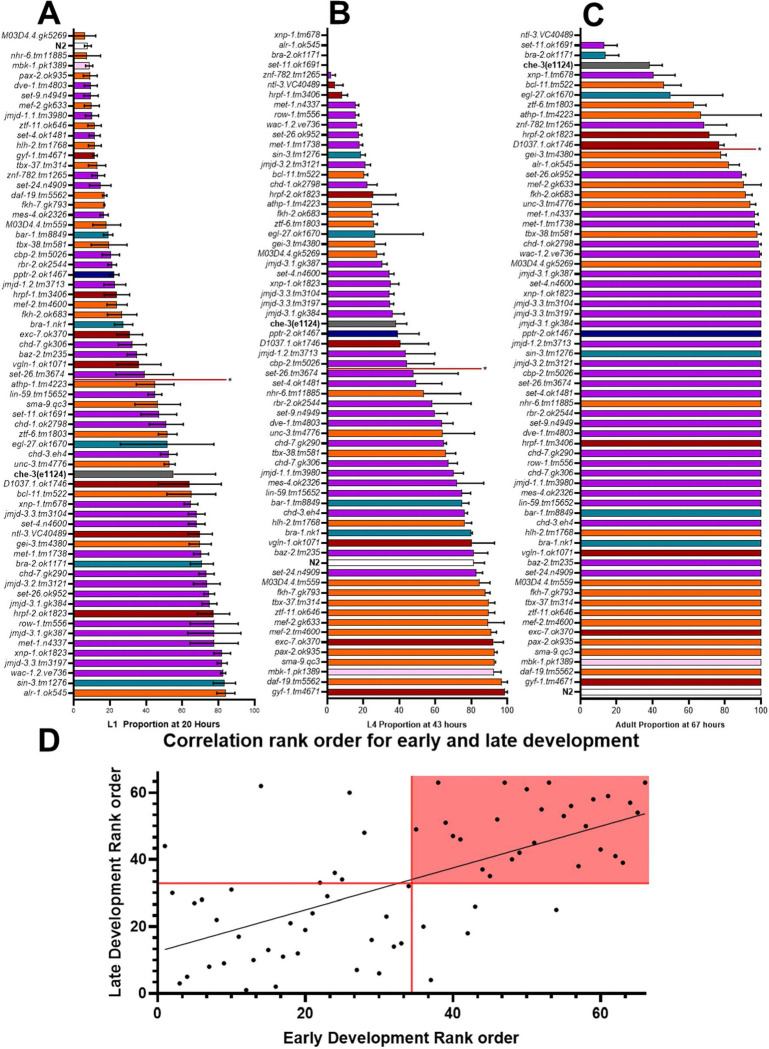
Gross developmental delays identified in ASD associated mutants vs N2 A. L1 proportion at 20 hours (approximate time of L1 moult to L2) of N2 compared to ASD associated mutants and chemosensory deficient mutant *che-3 (e1124)*. **B.** L4 proportion at 43 hours (approximate time of L3 moult to L4) of N2 compared to ASD associated mutants and *che-3 (e1124)* **C.** Adult proportion at 67 hours (Sufficient time for N2 worms to reach adulthood) of N2 compared to ASD associated mutants and *che-3 (e1124)*. n = 18 for N2 and n = 3 for all other mutants. All data shown as Mean±SEM. Statistical analysis performed using one-way ANOVA and Dunnetts’s multiple comparison test. Vertical red lines indicate significance compared to N2, anything above the red line is significantly different. Chromatin remodelers, transcription factors, RNA binding proteins, transcription co-factors, phosphatase, kinase. **D** Correlation of each strains developmental rank order identified by developmental rate analysis. Early development is defined as significant difference from wildtype (N2) at L1/L2 moult occurring at ~20 hours. Late development is defined as a significant difference from the wildtype (N2) at the L3/L4 moult time of ~43 hours or delayed reaching adulthood ~67 hours. The red lines mark the significance cutoff vs N2, and the red square highlights the area which is significantly different in both developmental categories.

Precise co-ordination of gene expression is important for tissue development and function. We measured the developmental progression of the selected autism associated mutant strains by recording the proportion of developmental stages at timepoints in the *C. elegans* developmental lifecycle that corresponds to larval moults during early, mid and late development. At 20 hours, the approximate time of the L1 moult to L2, the proportion of L1 worms was 7% for the N2 as the majority of the population had undergone the first larval molt to L2 (Fig 4A). The chemosensory deficient mutant, *che-3 (e1124)* displays significantly delayed development across all life stages. Thus, sensory deficits can give rise to developmental delays which are pervasive providing a benchmark for solely sensory based developmental delays. Conversely to the egg count analysis, there was no clear association between a specific molecular function and phenotypic output (Fig 3, 4 A,B,C).

Over half of the strains (34 of 65) investigated displayed an early developmental delay. A significant delay in early development was observed in the mutant strain *alr-1(ok545)* (Fig 4A), which had the highest proportion of L1s compared to N2 (12-fold increase). A significant delay in mid and late development was observed in 33 of the 65 strains (Fig 4B, 4C). Plotting a rank order comparison of early and late development for each strain can show how pervasive the developmental delays are. There was a high concordance of strains with significant early and late developmental delays. This is indicated by 80% of strains with an early developmental delay also satisfying the criteria for delayed later stages of development (Fig 4D). This shows the majority of strains fit the pattern observed in human cohorts of a pervasive developmental disorder which is present early in development and sustained (Minniscalco and Carlsson, 2022). However, 18% of the strains identified with a late developmental delay had no observed early developmental delay. Conversely, 15% that had an early developmental delay showed amelioration by the time these strains reached late development. In this case it implies potential compensatory mechanisms within these strains.

Finally, a large proportion encompassing 27 strains had no identified developmental delay. These results suggest that the two most common outcomes are pervasive developmental delays or strains with mutant autism associated genes that were unaffected in development.

### Neuroarchitectural changes in key sensory neurons are rare in ASD associated mutants

In the context of autism, the interaction between developmental programming and structural changes in the nervous system are well documented (Hashem et al., 2020). To address if there was a detectable difference we focussed on the core sensory structures that mediate the environmental cues. These are the amphid and phasmid structures. Given the growing emphasis of research on sensory systems in ASD, we wanted to develop the analysis around assays that quantified sensory responses to distinct modalities. These environmental inputs are mediated primarily by ASH for aversive modalities (Krzyzanowski et al., 2016) and AWA for diacetyl (Zhang et al., 1997). When dysfunctional, they underpin disrupted sensory responses. Before initiating this, we investigated the structural integrity of these sensory neurons, where possible, using DiI labelling.

**Fig 5.**
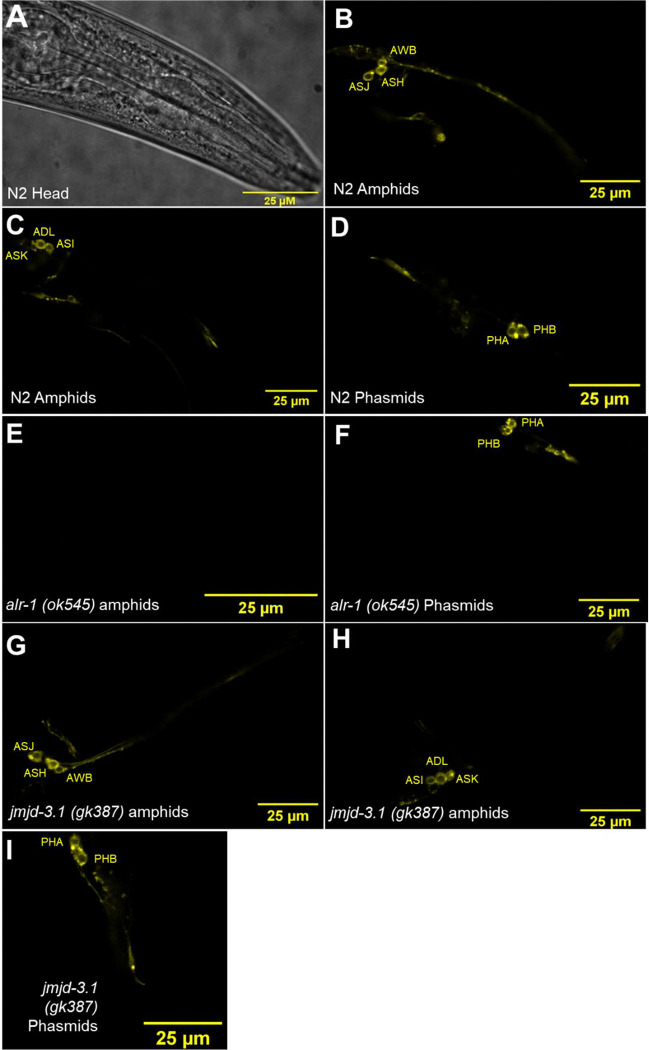
Sensory processing architecture in ASD associated *C. elegans* mutants. **A.** Brightfield image of N2 head showing the area imaged for analysing amphid presence. **B.** N2 sensory amphids, ASH, AWB and ASJ. **C.** N2 sensory amphids ASK, ADL and ASI. **D.** N2 sensory phasmids PHA and PHB. **E.** *alr-1(ok545)* missing sensory amphids. **F.** *alr-1(ok545)* showing presence of both PHA and PHB. **G.** *jmjd-3.1 (gk387)* sensory amphids ASJ, ASH and AWB. **H.** *jmjd-3.1 (gk387)* sensory amphids ASI, ADL and ASK. **I.** *jmjd-3.1 (gk387)* sensory phasmids PHA and PHB.

DiI staining was used to test for the presence of anatomical features primarily associated with sensory detection that lie upstream of the integrating and output aspect that co-ordinate integrated behaviour. As previously described, DiI was sufficient to stain the 6 amphid sensory neurones ASK, ASJ, ASH, ASI, AWB and ADL and both phasmids, PHA and PHB of the N2 strain (Fig 5A–C) (Collet et al., 1998). The same staining protocol was applied to mutants and then imaged for the presence or absence of amphids and phasmids (*che-3 (e1124)* used as a positive control).

Only two of the ASD associated mutants had a significant alteration to their amphid/phasmid structure, as previously described. These mutants were *alr-1 (ok545)* (Tucker et al., 2005) and *egl-27 (ok1670)* (Solari et al., 1999). *alr-1 (ok545)* showed a complete loss of dye filling by adulthood in the six sensory amphids (Fig 5D). However, this was selective as phasmids were still capable of dye filling (Fig 5E) in two of the six worms imaged. *egl-27 (ok1670)* showed the opposite pattern with both phasmids being unlabelled but all six sensory amphids accounted for. Thus, the gross sensory structures were unaffected in the majority of the strains when compared to N2. This is shown by *jmjd-3.1* (*gk387*) which showed all their amphids and phasmids (Fig 5 F,G,H) but as we describe later exhibited both pervasive developmental delays as well as sensory deficits. Based on this, the mutants in the pipeline showed no gross disruption in structures that initiate sensory processing, thus informing interpretation of subsequent behavioural analysis.

### ASD associated mutants cause hypo or hypersensitive behavioural responses to chemosensory stimuli

We evaluated sensory processing capabilities within each strain using chemoattractant and aversive cues. A diacetyl (1% v/v) quadrant assay (Fig 2) was utilised to measure attractive chemosensory processing. In this assay each worm had to detect the positive cue and integrate that signal into a locomotory response and move in the direction of the cue (chemotaxis). The observed chemotaxis of the mutants was compared to the N2 performance to determine any modified sensitivity to the attractive cue.

In addition, aversive chemosensory processing was assayed by quantifying reversals to both water soluble and volatile aversive cues. Sensory processing of attractive and aversive chemosensory cues requires the functionality of different sensory amphids (ASH or AWA), but downstream interneuron and motor neuron circuits are shared. Thus, by comparing strains across multiple modalities it is possible to distinguish between strains impacted at the level of sensory detection versus those impacted in downstream circuitry.

#### Chemotaxis response perturbed in 25% of ASD associated mutants

**Fig 6.**
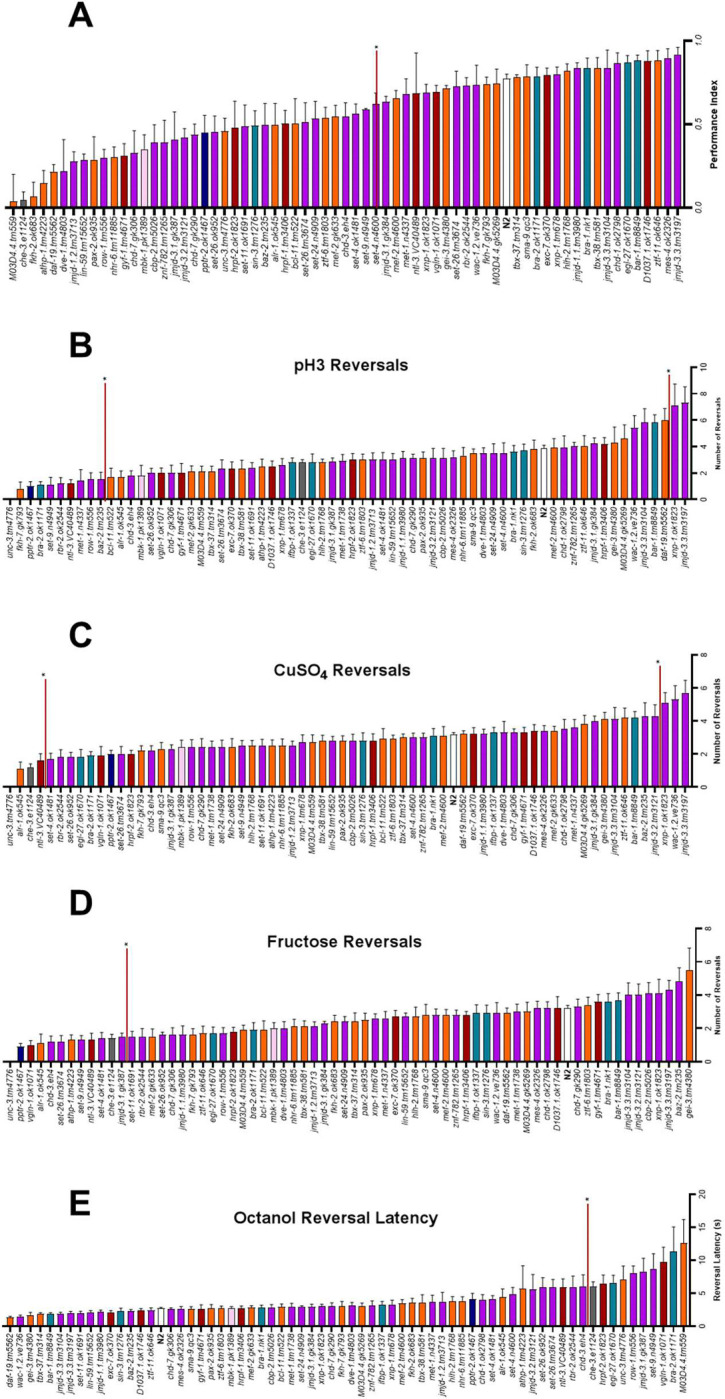
Sensory processing capabilities modified in *C*. *elegans* ASD associated mutants. **A.** Performance index of N2 vs ASD associated mutants in diacetyl quadrant assay. n=48 for N2. n = 3 for all other strains. Statistical analysis performed using one-way ANOVA and Dunnett’s multiple comparison test. **B, C, D.** Average number of reversals of N2 vs ASD associated mutants in response to aversive stimuli (low pH, copper sulphate and fructose). **E.** Latency to initiating reversals of N2 vs ASD associated mutants in response to 30% octanol. **B, C, D, E.** Statistical analysis performed using one-way ANOVA and Dunnett’s multiple comparison test. n = 180 for N2 and n = 10 for all other mutants. All data shown as Mean±SEM. Chromatin remodelers, transcription factors, RNA binding proteins, transcription co-factors, phosphatase, kinase. Significance shown by red horizontal line, in A and D strains above the red line are significant, in B. C and E strains outside of those contained with the red lines are significant vs N2.

N2 showed a reproducible attraction to the chemosensory cue diacetyl (1% v/v) as evidenced by the performance index of 0.77 (Fig 6A). 16 mutants showed a significant reduction in their performance index compared to N2. There was no clear bias for any of the molecular functional groups with each group represented proportionally. Additionally, only one strain, *M03D4.4 (tm559)*, showed a performance index lower than the negative control *che-3 (e1124)* of 0.05 with the remaining significantly impaired strains scoring between 0.07 and 0.42. This suggests an incomplete loss of sensory detection or sensory processing as most mutants fall between normal processing by N2 and complete loss of sensory processing by *che-3* (*e1124*).

#### Aversive cue responses show hypo and hypersensitivity

**Fig 7.**
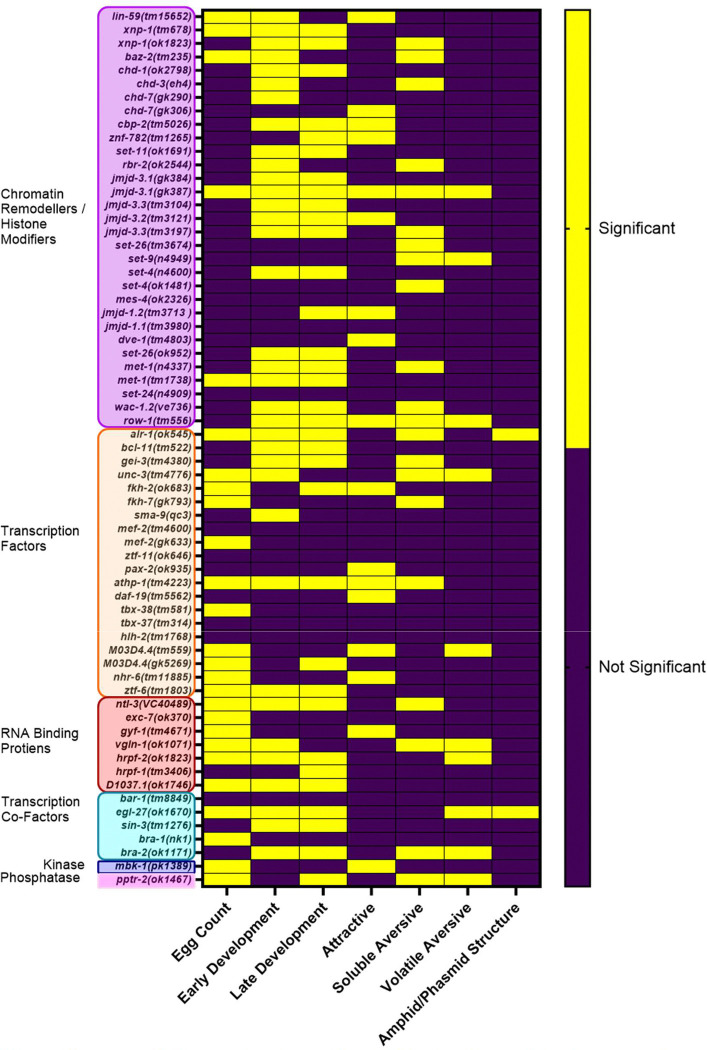
Significance heatmap of ASD associated mutant’s vs N2 for developmental and sensory phenotypes. Significance heatmap showing the seven phenotypes assessed in the experimental pipeline including, egg count, early and late development, attractive chemosensory processing, soluble aversive chemosensory processing, volatile aversive chemosensory processing and sensory amphid/phasmid structure. Yellow boxes show significant difference from N2, and purple are non-significant vs N2, in each respective phenotype. Significance is defined at p≤0.05. For egg count, early and late development, attractive, soluble aversive and volatile aversive chemosensory processing one-way ANOVAs and Dunnett’s multiple comparison test were performed to determine significance. For amphid/phasmid structure any deviation from the expected six amphids and two phasmids was noted as significant.

We measured responses to different aversive cues which utilise distinct molecular determinants to trigger the initiating response that drives the behaviour of reversing. In addition, we extended this to aversive volatile cues. Hypersensitivity, defined in this study as an enhanced aversive behavioural response compared to the wild-type was observed in *xnp-1* (*ok1823*), *jmjd-3.3* (*tm3197*) and *wac-1.2* (*ve736*) in response to low pH and copper sulphate (Fig 6B, 7C) and 1-octanol (Fig 6E). Hyposensitivity, defined as a reduced aversive behavioural response was exhibited by 17 mutants, to one or more of the soluble aversive cues. 6 of the 9 strains that were hyposensitive to 1-octanol demonstrated hyposensitivity to at least one soluble cue (Fig 6B–E).

Of the 65 strains tested 25 reached the criteria that indicate they show significant changes in response to the sensory cues (Fig 6B–E) compared to N2.

Hyposensitivity to the aversive cues was more commonly observed at 30 instances compared to just 5 instances of hypersensitivity.

### Significance heatmap of developmental and sensory assays of ASD associated mutants show functional segregation

**Fig 8.**
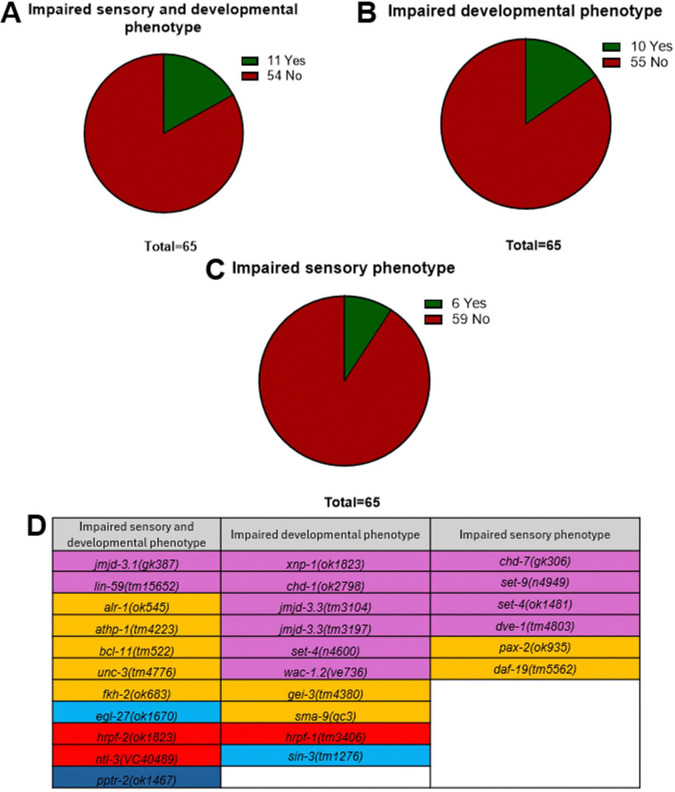
Filtering for significant developmental or sensory affected mutants highlights three distinct groupings. **A.** Subclassification of mutant strains for deficits in sensory processing, gross developmental delays and egg laying egg laying deficits. **B.** Phenotypically filtered genes for deficits in developmental programs and egg laying but without any sensory function deficits. **C.** Phenotypically grouped genes for deficits in sensory function without any gross developmental delays or fecundity deficits. **D.** Genes filtered from the significance heatmap for impaired sensory, impaired developmental and mixed phenotypes. The phenotypes grouped as developmental for the purpose of filtering were egg count, early development and late development. The phenotypes grouped as sensory were attractive chemosensory, soluble and volatile aversive chemosensory.

The results of the developmental, anatomical and sensory phenotypic analysis are summarised in figure 8 and 9. To aid in the identification of patterns within the dataset each mutant’s p value from each assay was converted to a binary value indicating significant or not significant and then plotted into the heatmap. Each row therefore is the specific phenotype observed in a particular strain and can be directly compared to other strains to highlight phenotypic overlap within the dataset. Furthermore, each gene is associated with a specific molecular functional grouping allowing for comparisons across individuals within a group as well as across groups.

Egg laying appears very sensitive to mutant perturbation, with over 50% of transcription factors and over 80% of RNA binding proteins showing significance compared to less than 20% of chromatin remodellers studied. Early and late developmental phenotypes are not associated with any one molecular functional grouping as evidenced by a consistent percentage of strains in each group showing developmental delays. Furthermore, there are no genes that are significantly affected across all phenotypes suggesting that there is no fully penetrant sensory deficient mutant which is also impaired in development and neuroarchitectural changes.

Across all strains analysed 89% had at least one phenotype (Fig S1A) leaving just 7 strains which had no phenotype across any of the assays used here. Gross changes to sensory structures was the least commonly identified phenotype affecting just 3% or 2 strains (*alr-1* and *egl-27)* both of which showed additional phenotypes in development and sensory processing. Developmental and sensory phenotypes were almost equally common in the strains investigated at 42 and 38 strains respectively (Fig S1A). Finally, sensory impairment overlapped with both developmentally delayed and non-delayed strains at a similar percentage but there is a prominent difference in the overlap of severe sensory impairment with developmental delay compared to non-delayed strains (Fig S1B).

### Three phenotypic groupings emerge from developmental and sensory screen of ASD associated mutants

The integration of our analysis from genes that emerged in our pipeline provided an opportunity to assess associations between the distinct domains from the analysis summarised above. We conducted a phenotypic grouping analysis to parse the cohort of mutants that passed through the comparative pipeline. This approach that integrates phenotypic score with each gene established 3 groups of genes. The classification was driven by ascribing normal or perturbed phenotypic response based on multiple comparison statistical tests of each gene in each assay (Statistical tests in figure legends). This is indicated on each graph and best reflected in the summary table (Fig 7) by a binary scoring in which a measured trait is defined as normal or perturbed.

The subgroups derived from Figure 8 were classified as being sensory deficient, developmental deficient or showing a combined sensory and developmental deficiency relative to controls. Group 1, deficient in both sensory responses, development and reproduction, includes 2 chromatin remodelers: 5 transcription factors and one cofactor, 2 RNA binding protein and a phosphatase (Fig 8 A, D). Group 2, impaired in only development, includes 6 chromatin remodelers, 2 transcription factors, one cofactor and an RNA binding protein (Fig 8 B, D). Group 3, impaired in only sensory responses, includes 4 chromatin remodelers and 2 transcription factors (Fig 8 C, D). Group 1 genes show a significant difference across all phenotypes investigated except neuroarchitectural changes which provides a subset of genes with pleiotropic effects. Group 2 and 3 genes show more selective effects in development and sensory processing respectively.

## Discussion

A widely recognised phenotypic trait associated with autism is the atypical sensory processing. This is readily observed in clinical cohorts and has emerged as a listed characteristic in the updated DSM V (American Psychiatric Association, 2013). This supplements the established criteria around social and repetitive motor behaviours and the signature early life emergence of the neuroatypical traits. In clinical cohorts the reported changes span multiple sensory modalities from visual, auditory to tactile and chemosensory (Sivapalan et al., 2024; Bennetto et al., 2007), although they are not systematically recorded. However, individuals with ASD have shown an increased severity in behavioural issues because of sensory processing changes requiring routine therapy to try and alleviate the problematic behaviours (Sivapalan et al., 2024). This highlights the severity of real-world consequences for individuals with ASD and the lack of genetic or pharmacological targets available to ameliorate the changes.

This recognized significance of the sensory component led us to design a pipeline in *C*. *elegans* that sort to exploit approaches that inform on this aspect. We designed a pipeline to resolve epigenetic and wider genetic modulators in which there is good evidence for intersection with environmental cues that go through sensory pathways. In addition, this pipeline utilizes epigenetic regulators that contribute to polygenic transcriptional programs that may represent a distributed polygenic landscape like those that represent the most significant genetic determinants of autism. The pipeline is supplemented by additional transcriptional regulators. In restricting our analysis to category 1 SFARI genes it allowed the comparison of key biological traits encompassing developmental and sensory responses in the highest confidence gene expression modifiers.

Furthermore, developmental delays in ASD cohorts have correlated with a greater likelihood of other ASD symptoms such as impairments in fine motor skills, language ability and personal social activities (Shan et al., 2022). For this analysis, it is important to note that intrinsic changes in gross development represented in the mutations are confounded by the potential for gross sensory disruption which is sufficiently penetrant to execute developmental changes as exemplified by the positive control *che-3 (e1124).* Therefore, understanding the intersection of developmental processes and sensory processing changes in ASD is vital to develop pharmacological treatments.

When investigating bioinformatically selected autism associated genes, we identified that development and by extension underpinning programs were especially susceptible to perturbation with 61% of strains being impacted in either early or late development. Between early and late a number of subtle transitions occur between early larval stages but, by their fourth molt, 400 epidermal, gonad, muscle and neuronal cells have been added to a total of 959 somatic cells (Rougvie and Moss, 2013). This process is susceptible to perturbation at any point which could lead to a developmental delay.

Mutants that showed perturbations early in development were largely pervasive with over 80% concordance of strains with significant early/late developmental delays suggesting delays in early development were not easily overcome. However, this was not universal and 15% of strains with an early developmental delay showed no significant delay in later development, suggesting a possible mechanism by which these strains were able to expedite their later development. This is not unprecedented as early perturbation of developmental programs have been corrected by late expression of the affected gene (Luikenhuis et al., 2004).

An association to the human disorder can be made here as there have been documented examples of spontaneous recovery from an autistic phenotype suggesting the possibility that recovery of developmental delays is not strictly a worm phenotype (Sitholey et al., 2009). Another example is MeCP2 the main driver of Rett’s Syndrome (RTT) in which expression of functional MeCP2 in mutant mice lines rescue the RTT phenotype (Luikenhuis et al., 2004). Despite this phenotypic rescue originating from transgenic expression of the affected gene, it does not preclude the possibility for endogenous mechanisms being present within *C. elegans* that are capable of self-rescuing developmental delays. Additionally, environmental cues from other worms on the same plate have been shown to affect developmental rates, providing an example of environmental impact on development which could also contribute to the developmental phenotypes we observed (Ludewig et al., 2017).

The criteria used to define sensory dysfunction was based on being significantly perturbed compared to N2 in either attractive, soluble aversive or volatile aversive chemosensory processing. However, when considering mutants that had impaired development, the potential impact becomes clearer when highlighting a more severe sensory phenotype. Increased severity here is defined as significant perturbations across multiple sensory modalities. Severity of sensory perturbation overlaps with developmental delays. This adds credence to our approach of scoring development alongside behavioural outcomes as 27.5% of strains with an impairment in early or late development show an increase in severity of impact to sensory processing.

Interestingly, it is only the severity of the sensory perturbations which correlates with developmental delays, as developmentally delayed strains had a 55% overlap with sensory perturbations compared to 60% of non-developmentally delayed strains. This highlights that the worm has a distinct relevance to modelling this condition as the same effect of developmental delays correlating with greater severity of ASD symptoms in human cohorts (Shan et al., 2022). Furthermore, it shows that sensory perturbations in the adult can arise without a developmental delay being present, highlighting the possibility for selectivity in the genetic underpinnings of different subsets of ASD mutants.

Our analysis led to three groups of mutants which can be aligned with dysfunction in different areas of ASD symptomology investigated, namely Group 1: sensory, gross development and reproduction; Group 2: gross development and reproduction; Group 3: sensory. These sub-groups which are criteria-based, contain orthologues spanning diverse molecular functions. One speculation is that these represent distinct but definable grouping in which the flagged molecular perturbation generates shared mechanistic behaviours. This view expresses outcomes from genetic interrogation of symptomology from human cohorts (Litman et al., 2025; Leyhausen et al., 2025). These studies highlight the preferred view that distinct subcategories relate to distributed mechanistic underpinnings (Litman et al., 2025; Leyhausen et al., 2025). Our criteria-based approach in which the intrinsic developmental programs and associated behavioural outcomes in the adult do a similar thing in a biologically simpler organism demonstrates the capacity to model the ASD spectrum in *C. elegans*. This provides a basis for further analysis to investigate the transcriptional profile within each grouping identified in our study to determine if shared mechanistic pathways underpin behavioural changes within each group and differentiate the groups from each other.

These notions are consistent with a recent studies evidence for selective pathways altered in ASD individuals creating a unique presentation of the disorder (Litman et al., 2025). However, one understandable limitation in these human cohort studies is the absence of sensory phenotypes in any of the groups they identify. The preferred interpretation of our data supports the need for a more systematic sensory testing of ASD cohorts. This would require quantitative sensory testing in ASD cohorts with known genetic backgrounds. Using the 3 gene groups segregated from our investigation we attempted to identify sensory phenotypes in human and mouse cohorts but due to limited sensory information it was difficult to determine if our identified genes correlated with human or murine findings.

In conclusion, investigation of ASD associated gene expression modifier orthologues in *C. elegans* led to a criteria-based grouping with shared phenotypic disruptions. These groupings encompass genes with distinct divergent biological functions with a shared ability to modulate wider gene expression. We drove this by considering the phenotypic overlap between developmental timing and quantifiable and systematic measurement of sensory processing. The latter is an important and underreported component of ASD pathology with a direct impact upon an individual’s behaviour and quality of life (Giacardy et al., 2018). Therefore, further investigation into the molecular basis of sensory processing perturbation is necessary and our robust experimental pipeline can identify candidate genes for further analysis within this proposed framework.

## Supplementary Material

Supplement 1
