# Supplementary material for: Sensory and developmental phenotyping of C. elegans parses autism associated genes into behavioural classifications
Source: bioRxiv. 2026 Mar 30:2026.03.27.714775. Preprint. [Version 1] doi: 10.64898/2026.03.27.714775 (PMC13060289; doi:10.64898/2026.03.27.714775)
Supplement: Supplement 1 [file media-1.pdf]

Human gene	Gene name	Protein function	*C. elegans* ortholog ue	Allele	Strain name	Outcros sed	Mutation
ASH1L	ASH1 like histone lysine methyltransfe rase	Histone methyltransfe rase	*lin-59*	tm1565 2	FX25652	x0	Deletion
ATRX	ATRX chromatin remodeler	SWI/SNF chromatin remodeler	*xnp-1*	tm678	FX00678	x0	Deletion
				ok1823	VC2088	x1	Deletion
BAZ2B	Bromodomain adjacent to zinc finger domain 2B	Component of chromatin remodeling complexes	*baz-2*	tm235	FX00235	x0	Deletion
CHD2	Chromodoma in helicase DNA binding protein 2	SNF2 related chromatin remodeler	*chd-1*	ok2798	RB2613	x0	Deletion
CHD3	Chromodoma in helicase DNA binding protein 3	SNF2 related chromatin remodeler	*chd-3*	eh4	HX103	x4	Deletion
CHD8	Chromodoma in helicase DNA binding protein 8	SNF2 related chromatin remodeler	*chd-7*	gk290	VC606	x0	Deletion
				gk306	VC676	x0	Deletion
CREBBP	CREB binding protein	Histone acetyltransfer ace and transcriptiona l regulator	*cbp-2*	tm5026	FX05026	x0	Deletion
CTCF	CCCTC-binding factor	Component of histone acetylase and deacetylase complexes and transcriptiona l regulator	*znf-782*	tm1265	FX01265	x0	Deletion +Insertion
EHMT1	Euchromatic histone lysine methyltransfe rase 1	Histone methyltransfe rase	*set-11*	ok1691	VC1219	x0	Deletion
KDM5B	Lysine demethylase 5B	Jumonji/ARID family lysine demethylase	*rbr-2*	ok2544	RB1941	x0	Deletion
KDM6B	Lysine demethylase 6B	Jumonji/ARID family lysine demethylase	*jmjd-3.1*	gk384	VC936	x0	Deletion + Insertion
				gk387	VC912	x0	Deletion

			jmjd-3.2	tm3121	FX03121	x0	Deletion + Insertion
			jmjd-3.3	tm3104	FX03104	x0	Deletion
			jmjd-3.3	tm3197	FX34427	x2	Deletion
KMT2E	Lysine methylransfer ase 2E	Histone methyltransfe rase	*set-26*	tm3674	FX03674	x0	Deletion
KMT2E			*set-9*	n4949	MT1642 6	x1	Deletion
KMT5B	lysine methylransfer ase 5B	Histone methyltransfe rase	*set-4*	n4600	MT1491 1	x2	Deletion
KMT5B				ok1481	VC997	x0	Deletion
NSD1	Nuclear receptor binding SET domain protein 1	Histone methyltransfe rase	*mes-4*	ok2326	VC1874	x1	Deletion + Insertion
PHF2	PHD finger protein 2	Histone demethylase	*jmjd-1.1*	tm3980	FX17844	x2	Deletion
PHF2			*jmjd-1.2*	tm3713	FX17361	x2	Deletion
SATB1	SATB Homeobox 1	Chromatin remodeling scaffold	*dve-1*	tm4803	FX04803	x0	Deletion
SETD1A	SET domain containing 1A	Histone lysine methyltransfe rase	*set-26*	ok952	RB1025	x0	Deletion
SETD2	SET domain containing 2	Histone lysine methyltransfe rase	*met-1*	n4337	MT1697 3	x4	Deletion
SETD2				tm1738	FX01738	x0	Deletion
SETD5	SET domain containing 5	Histone lysine methyltransfe rase	*set-9*	n4949	MT1642 6	x1	Deletion
SETD5			*set-24*	n4909	MT1613 3	x0	Deletion
WAC	WW domain containing adaptor with coiled-coil	Signalling protein	*wac-1.2*	ve736	RG3236	x0	Deletion
POGZ	Pogo transposable element derived with ZNF domain	Chromatin remodeling complex protein and transcription factor	*row-1*	tm556	FX00556	x0	Deletion
ARX	Aristaless related homeobox	Homeobox transcription factor	*alr-1*	ok545	RB762	x0	Deletion
BCL11A	BCL11 Transcription Factor A	Transcription factor	*bcl-11*	*tm522*	FX0522	x0	Deletion
CIC	Capicua Transcription al Repressor	Transcription al repressor	*gei-3*	*tm4380*	FX04380	x0	Deletion
EBF3	EBF Transcription Factor 3	Transcription al activator	*unc-3*	*tm4776*	FX04776	x0	Deletion
FOXG1	Forkhead Box G1	Transcription al repressor	*fkh-2*	*ok683*	RB853	x0	Deletion

FOXP1	Forkhead Box P1	Transcriptional repressor	*fkh-7*	*gk793*	VC1646	x0	Deletion
FOXP2	Forkhead Box P2	Transcriptional repressor					
HIVEP2	HIVEP Zinc Finger 2	Transcriptional activator	*sma-9*	*qc3*	CS67	x1	Point Mutation (Stop Gained)
MEF2C	Myocyte Enhancer Factor 2C	Transcriptional activator	*mef-2*	*tm4600*	FX04600	x0	Deletion
MEF2C				*gk633*	VC1402	x0	Deletion
MYT1L	Myelin Transcription Factor 1 Like	Transcription factor	*ztf-11*	*ok646*	RB824	x0	Deletion
PAX5	Paired Box 5	Transcription factor	*pax-2*	*ok935*	RB1013	x0	Deletion
PHF12	PHD Finger Protein 12	Transcriptional repressor	*athp-1*	*tm4223*	FX04223	x0	Deletion
RFX3	Regulatory Factor X3	Transcription factor	*daf-19*	*tm5562*	FX05562	x0	Deletion
TBR1	T-Box Brain Transcription Factor 1	Transcriptional repressor	*tbx-38*	*tm581*	FX0581	x0	Deletion
TBR1			*tbx-37*	*tm314*	FX0314	x0	Deletion
NR4A2	Nuclear Receptor Subfamily 4 Group A Member 2	Transcriptional regulator	*nhr-6*	*tm11885*	FX011885	x0	Deletion
TCF4	Transcription Factor 4	Transcription factor	*hlh-2*	*tm1768*	FX01768	x0	Deletion
VEZF1	Vascular Endothelial Zinc Finger 1	Transcription factor	*M03D4.4*	*tm559*	FX0559	x0	Deletion
VEZF1				*gk5269*	VC4183	x0	Deletion (Balanced)
ZBTB20	Zinc Finger And BTB Domain Containing 20	Transcription factor	*ztf-6*	*tm1803*	FX01803	x0	Deletion
CNOT3	CCR4-NOT Transcription Complex Subunit 3	mRNA deadenylase	*ntl-3*	*gk944863*	VC40489	x0	Deletion
ELAVL3	ELAV Like RNA Binding Protein 3	RNA-binding protein	*exc-7*	*ok370*	VC176	x0	Deletion
GIGYF2	GRB10 Interacting GYF Protein 2	Repressor of translation initiation	*gyf-1*	*tm4671*	FX04671	x0	Deletion
HDLBP	High Density Lipoprotein Binding Protein	RNA-binding protein	*vgln-1*	*ok1071*	RB1093	x0	Deletion
HNRNPH2	Heterogeneous Nuclear Ribonucleoprotein H2	Heterogeneous nuclear ribonucleoprotein	*hrpf-2*	*ok1823*	VC2088	x1	Deletion
HNRNPH2			*hrpf-1*	*tm3406*	FX03406	x0	Deletion

SON	SON DNA And RNA Binding Protein	mRNA splicing cofactor	*D1037.1*	*ok1746*	RB1489	x0	Deletion
CTNNB1	Catenin Beta 1	Transcription cofactor	*bar-1*	*tm8849*	FX08849	x0	Deletion
IRF2BPL	Interferon Regulatory Factor 2 Binding Protein Like	Transcription corepressor	*ifbp-1*	*ok1339*	VC812	x0	Deletion
RERE	Arginine-Glutamic Acid Dipeptide Repeats	Transcriptional corepressor	*egl-27*	*ok1670*	VC1217	x1	Deletion
SIN3A	SIN3 Transcription Regulator Family Member A	Transcriptional corepressor	*sin-3*	*tm1276*	FX01276	x0	Deletion
ZMYND8	Zinc Finger MYND-Type Containing 8	Transcriptional corepressor	*bra-1*	*nk1*	NU1	x10	Deletion
ZMYND8			*bra-2*	*ok1171*	VC869	x1	Deletion (Balanced)
DYRK1A	Dual Specificity Tyrosine Phosphorylation Regulated Kinase 1A	CTD kinase of RNAP II	*mbk-1*	*pk1389*	EK228	x6	Deletion
PPP2R5D	Protein Phosphatase 2 Regulatory Subunit B'Delta	Ser/Thr Phosphatase	*pptr-2*	*ok1467*	RB1338	x0	Deletion

Table S1. *C. elegans* strain information

All ASD associated mutant strains used in this study as well as additional information including human gene name, protein function, *C. elegans* orthologue, allele, strain name, number of times outcrossed and mutation type. Colour scheme is as follows: Green – chromatin remodellers/ histone modifiers, Orange – transcription factors, Red – RNA binding proteins, Light Blue – transcription co-factors, Dark Blue – Kinase, Yellow – Phosphatase.

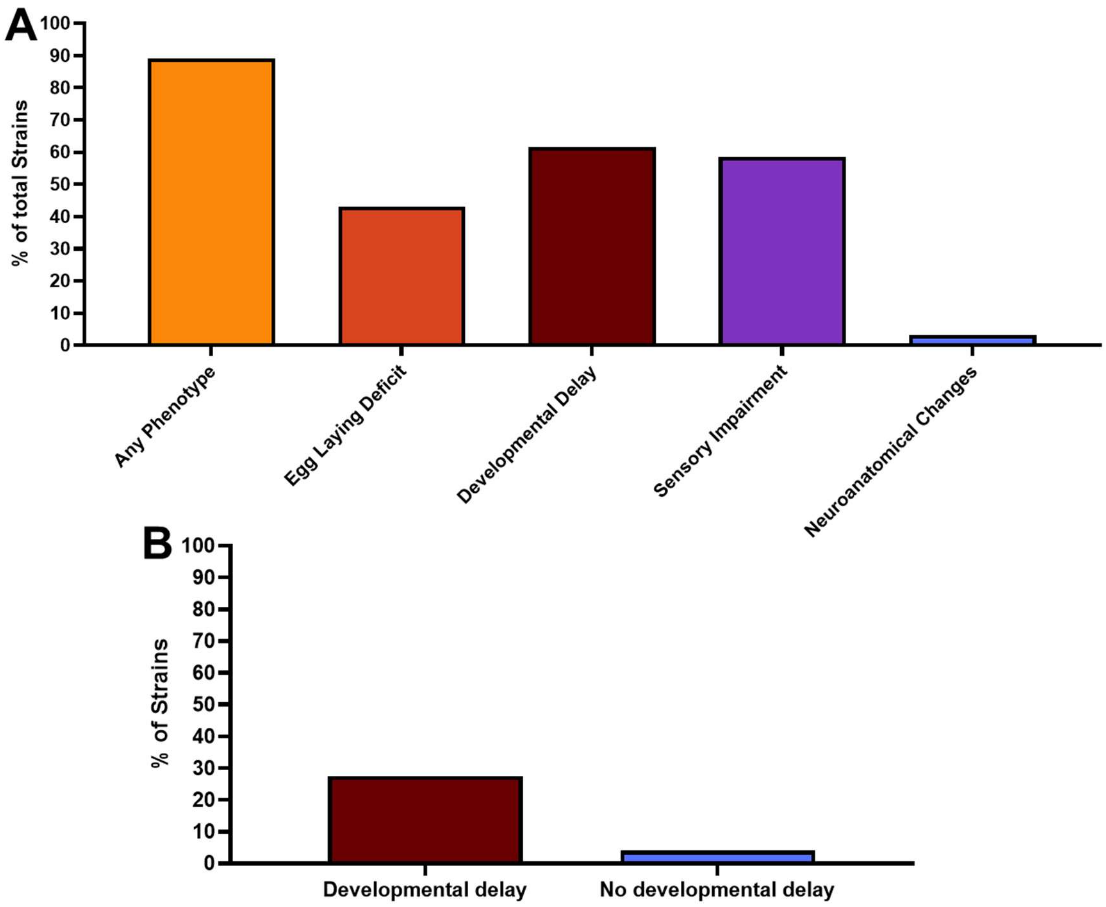

Fig S1. Phenotypic summary of all strains investigated.

A. Based on the percentage of strains with a given phenotype as outlined in figure 8. Each strain which is statistically different from N2 in any phenotype is represented under any phenotype. See methods for breakdown how each group is determined for the calculation of the % of total strains. **B.** Percentage of developmentally delayed strains with a severe sensory phenotype (See methods) compared to the percentage of strains with no developmental phenotype paired with a severe sensory phenotype.
